# The peripheral neuroimmune system

**DOI:** 10.1093/jleuko/qiae230

**Published:** 2024-10-18

**Authors:** Keaton Song, Brian S Kim

**Affiliations:** Graduate School of Biomedical Sciences, Icahn School of Medicine at Mount Sinai, One Gustave L. Levy Place, New York, NY 10029, USA; Nash Family Department of Neuroscience, Icahn School of Medicine at Mount Sinai, One Gustave L. Levy Place, New York, NY 10029, USA; Friedman Brain Institute, Icahn School of Medicine at Mount Sinai, One Gustave L. Levy Place, New York, NY 10029, USA; Kimberly and Eric J. Waldman Department of Dermatology, Icahn School of Medicine at Mount Sinai, One Gustave L. Levy Place, New York, NY 10029, USA; Marc and Jennifer Lipschultz Precision Immunology Institute, Icahn School of Medicine at Mount Sinai, 1425 Madison Ave, New York, NY 10029, USA; Mark Lebwohl Center for Neuroinflammation and Sensation, Icahn School of Medicine at Mount Sinai, 787 11th Ave, New York, NY 10019, USA; Allen Discovery Center for Neuroimmune Interactions, Icahn School of Medicine at Mount Sinai, 787 11th Ave, New York, NY 10019, USA; Friedman Brain Institute, Icahn School of Medicine at Mount Sinai, One Gustave L. Levy Place, New York, NY 10029, USA; Kimberly and Eric J. Waldman Department of Dermatology, Icahn School of Medicine at Mount Sinai, One Gustave L. Levy Place, New York, NY 10029, USA; Marc and Jennifer Lipschultz Precision Immunology Institute, Icahn School of Medicine at Mount Sinai, 1425 Madison Ave, New York, NY 10029, USA; Mark Lebwohl Center for Neuroinflammation and Sensation, Icahn School of Medicine at Mount Sinai, 787 11th Ave, New York, NY 10019, USA; Allen Discovery Center for Neuroimmune Interactions, Icahn School of Medicine at Mount Sinai, 787 11th Ave, New York, NY 10019, USA

**Keywords:** itch biology, mast cell, neuroimmunology, neuropeptide, sensory biology

## Abstract

Historically, the nervous and immune systems were studied as separate entities. The nervous system relays signals between the body and the brain by processing sensory inputs and executing motor outputs, whereas the immune system provides protection against injury and infection through inflammation. However, recent developments have demonstrated that these systems mount tightly integrated responses. In particular, the peripheral nervous system acts in concert with the immune system to control reflexes that maintain and restore homeostasis. Notwithstanding their homeostatic mechanisms, dysregulation of these neuroimmune interactions may underlie various pathological conditions. Understanding how these two distinct systems communicate is an emerging field of peripheral neuroimmunology that promises to reveal new insights into tissue physiology and identify novel targets to treat disease.

## Teaser

The peripheral neuroimmune reflex arc illustrates how immune cells can simultaneously respond to both motor and sensory nerves but also transmit signals to sensory neurons.

## Key Concepts

- Sensory and autonomic physiology extends into the immune system to regulate tissue homeostasis in peripheral organs.
- The cholinergic anti-inflammatory pathway defines a neuroimmune circuit by which the autonomic nervous system regulates systemic inflammation.
- Itch neuroimmunology has revealed that there are shared signaling pathways between sensory neurons and immune cells, suggesting that there is evolutionarily conserved crosstalk across the nervous and immune systems.

## Open Questions

- What key neuroimmune reflexes are triggered by the immune system to promote homeostasis?
- To what extent is the sensory arc the primary mediator of autonomic inflammatory pathologies?
- How overlapping are the functions of the vagal and spinal sensory nervous systems in regulating immunity and inflammation?

## 1. Introduction

The brain is considered the central organ that senses the environment and executes motor functions in conjunction with cognitive processing. It was originally believed to be separate from the rest of the body based on early experiments that showed that a dye injected into the bloodstream led to staining in all tissues except for the brain and spinal cord, which together comprise the central nervous system (CNS).^1^ Now, it is well appreciated that the blood–brain barrier, previously considered an impermeable passageway, is highly permissive to the trafficking of immune cells from the periphery to the brain. Further, tissue-resident immune cells such as microglia play critical roles in promoting brain health.^2^ Beyond the CNS, however, the new and emerging field of peripheral neuroimmunology is revealing how unique interactions between neurons and immune cells in various organs can profoundly impact mammalian health.

**Fig. 1.**
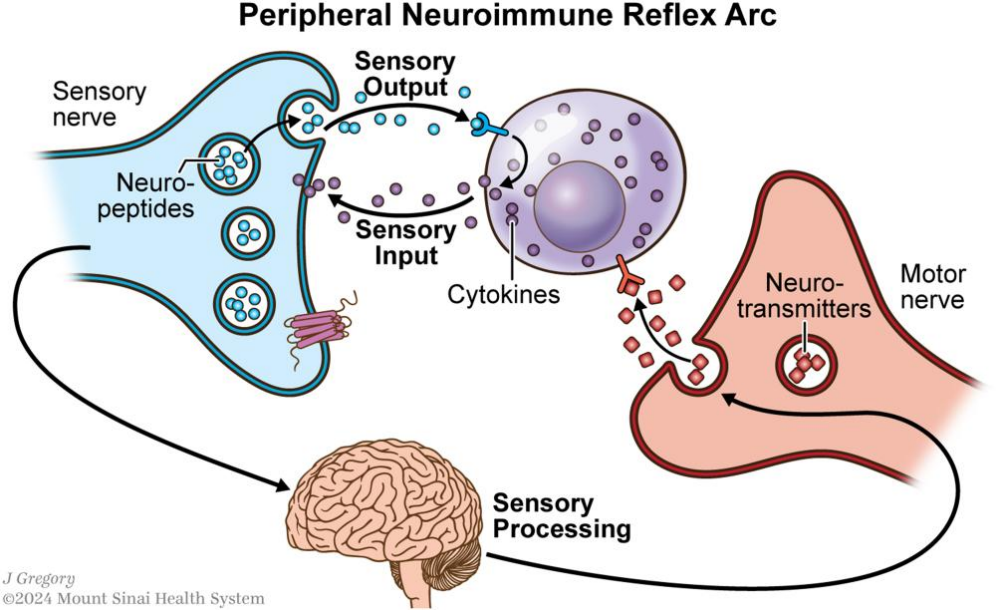
The peripheral neuroimmune reflex arc. Peripheral neurons are classified as autonomic (motor) neurons that release neurotransmitters, or as sensory neurons that respond to neurotransmitters and inflammatory factors like cytokines. Classical neurotransmitters from autonomic nerves can directly activate immune cells. Further, sensory neurons, in addition to receiving signals in afferent fashion, can also release neuropeptides that directly modulate immune cells. Ultimately, the brain perceives sensory signals and initiates efferent motor responses. The combined sensory and motor properties of the PNS and its combined capacity to regulate immune cells is what we refer to as the peripheral neuroimmune reflex arc.

Peripheral neuroimmunology focuses on cellular crosstalk between the peripheral nervous system (PNS), which includes all nerves outside of the CNS, and the immune system. Classically, it was thought that neurons and immune cells utilized distinct signaling pathways within their systems using neurotransmitters or cytokines, respectively. However, it is now known that there is convergence between these systems given that receptors for canonical neurotransmitters and cytokines are interchangeably expressed across neurons and immune cells (Fig. 1).^3–7^ This review will provide an overview of mechanisms by which the PNS, comprised of the autonomic and sensory nervous systems, interface with the immune system and highlight recent studies that have demonstrated how these interactions showcase the emerging frontier field of peripheral neuroimmunology.

## 2. Overview of the Peripheral Nervous System

**Fig. 2.**
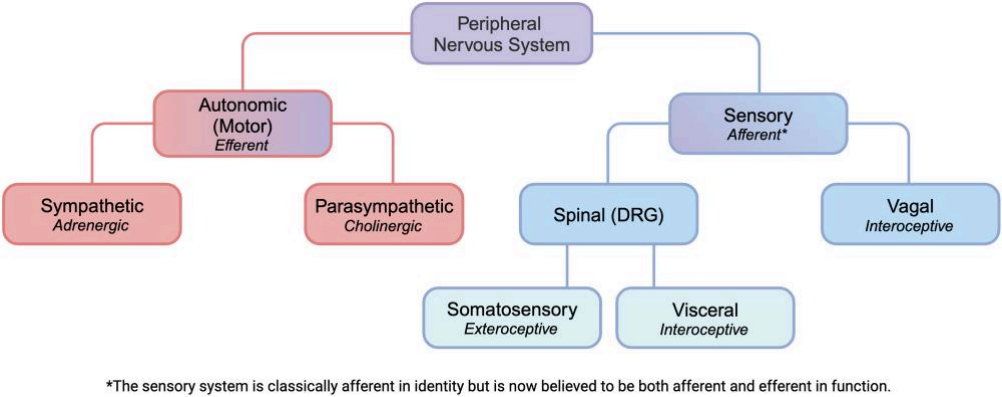
Classification of the peripheral nervous system (PNS). The PNS is functionally divided into the ANS and the sensory nervous system. The ANS is motor in function through its release of adrenergic and cholinergic neurotransmitters in an efferent manner. In contrast, the sensory nervous system is classically known to relay afferent signals to the brain. The sensory nervous system is broadly divided into the spinal sensory nervous system, which houses its cell bodies in DRG, and the vagal sensory nervous system, which houses its cell bodies in the jugular and nodose ganglia. The somatosensory branch of spinal nerves is considered exteroceptive in that it innervates target tissues that lie towards the surface of the body (e.g. skin). In contrast, visceral spinal nerves are considered interoceptive as they innervate inner organs. The vagus nerve, which largely innervates internal organs, is thus considered interoceptive as well. Though afferent in identity, it is now being revealed that sensory neurons can function in an efferent manner through the release of neuropeptides as well. Created with BioRender.com.

The PNS communicates extensively with the immune system to maintain homeostasis in the body, disruption of which can lead to various pathologies. It is composed of nerves as well as clusters of cell bodies of neurons called ganglia, all of which lie outside the brain and spinal cord. They transmit sensory information about the internal and external environments to the CNS. Subsequent to sensory stimulation, motor commands are generated in the cortex and relayed back to peripheral effectors via motor neurons, thus providing a means of communication between the brain and periphery. The PNS has two broad divisions: the autonomic nervous system (ANS) and the sensory nervous system (Fig. 2).

### 2.1 Autonomic nervous system

The ANS controls involuntary physiologic processes such as heart rate, blood pressure, respiration, and digestion. Neuronal signaling in this system is unidirectional; preganglionic neurons synapse on postganglionic neurons in sympathetic or parasympathetic ganglia, which represent two further divisions of the ANS. From there, postganglionic neurons innervate and release neurotransmitters to different target sites in peripheral tissues.

The sympathetic system regulates the adrenergic “fight-or-flight” response through the release of norepinephrine (NE). It arises from the thoracic and lumbar spinal cord segments and synapses on sympathetic ganglia to relay signals to tissues.^8^ In contrast, the parasympathetic system mediates the cholinergic “rest-and-digest” response via acetylcholine (ACh). Parasympathetic nerve fibers, a majority of which are associated with the vagus nerve, also arise from the sacral spinal cord and synapse on terminal ganglia that are proximal to the targets to which ACh is released.^9^ Most organs receive both sympathetic and parasympathetic innervations, whose opposing effects help maintain physiologic equilibrium. For example, activation of the sympathetic system leads to an increase in heart rate and blood pressure whereas activation of the parasympathetic system decreases both. This balance is lost in pathological conditions such as hypertension or irritable bowel syndrome, demonstrating that autonomic balance is crucial for the maintenance of homeostatic physiology.^10,11^ The field of peripheral neuroimmunology is suggesting that the ANS also helps preserve immune homeostasis and that adrenergic and cholinergic physiology extends deep into the immune system.

### 2.2 Sensory nervous system

**Fig. 3.**
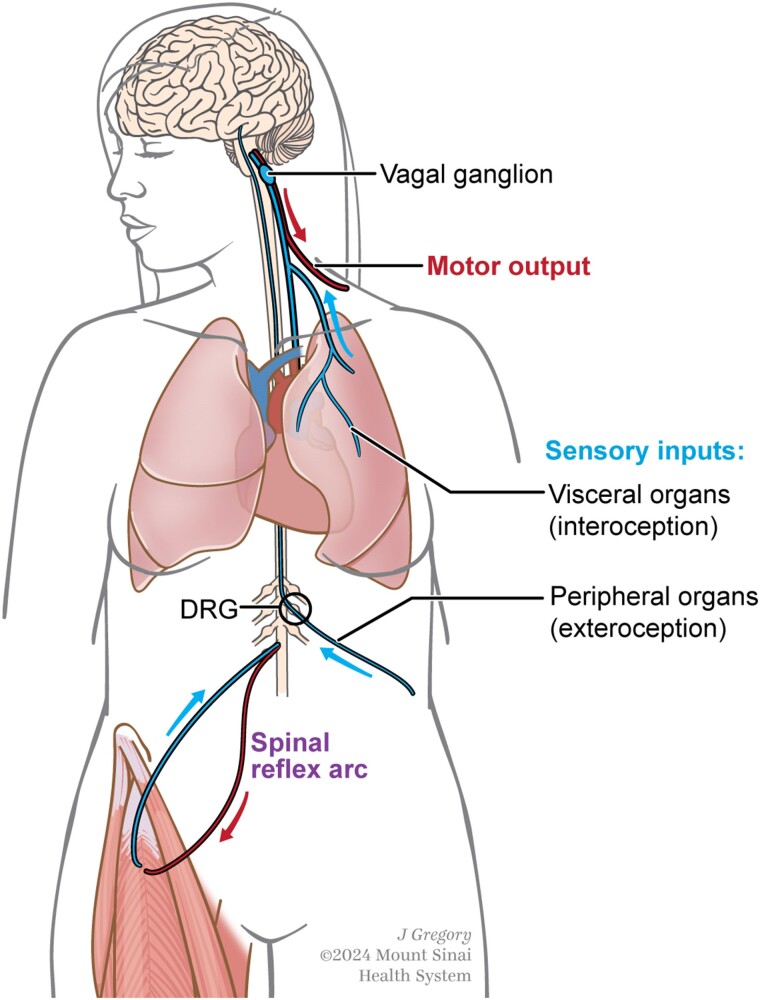
Organization of nerves comprising the reflex arc. All reflexes consist of a sensory component and a motor component. A well-known reflex arc is the spinal reflex, in which sensory neurons activate spinal motor neurons to generate movement without use of the brain. Other reflexes, such as those that get sensory input from peripheral and visceral organs, require sensory information to be transmitted to the brain in order to generate a motor output. The cell bodies of sensory neurons that relay such information are housed in the DRG or in vagal ganglia. The majority of DRG neurons innervate peripheral organs whereas a minority innervate visceral organs. Vagal sensory neurons only innervate visceral organs such as the heart, lungs, and gut. Upon receipt of sensory information, motor commands are generated in the brain and relayed to motor neurons, resulting in muscle movement.

In order for the aforementioned autonomic motor functions to take place, sensory information must first be transmitted from the PNS to the CNS (Fig. 3). The sensory nervous system is responsible for the perception of touch, pressure, proprioception, temperature, and pain. Sensory neurons are pseudounipolar: they have a single axon that bifurcates into two processes, one proximal (to the spinal cord or brainstem) and one distal (to the periphery). The cell bodies of primary sensory neurons that innervate the body are embedded within the dorsal root ganglion (DRG), which lies lateral to the spinal cord.^12^ Their detection of external stimuli activates G protein-coupled receptors (GPCRs) and modulates the activity of ion channels such as transient receptor potential (TRP) channels.^13^ A resultant membrane depolarization triggers the opening of voltage-gated sodium channels including Na_v_1.7, Na_v_1.8, and Na_v_1.9, and produces action potentials which sensory neurons transmit to the spinal cord and onto the brain to generate sensory perception.^14^ Though primarily studied for their afferent role in transmitting sensory information, it is now appreciated that sensory neurons also function in an efferent manner through their release of neural mediators, or neuropeptides.

Spinal sensory neurons are most well recognized for their classification as somatosensory neurons which comprise the largest sensory system in mammals and extensively innervate external organs such as the skin and soft tissues.^15^ Therefore, the somatosensory nervous system is often considered exteroceptive (sensing external cues). It is important to underscore that a sizable minority of DRG neurons also innervate visceral organs and are thus considered interoceptive (sensing internal cues).^16^ Understanding the unique and conserved features of these different spinal sensory neurons in the context of neuroimmune interactions is a burgeoning area of inquiry.

Vagal sensory neurons comprise the other major arm of the sensory nervous system.^17^ Their cell bodies are located in one of two vagal ganglia that make up the vagus nerve. Despite its primary association with the parasympathetic branch of the ANS, much of the vagus nerve contains sensory neurons. Like the aforementioned minority of spinal sensory neurons, vagal sensory neurons are thought to be interoceptive due to their innervation of visceral organs. Furthermore, although their primary physiologic functions are in sensing cardiac and respiratory signals, there is an increasing appreciation for their role in regulating a variety of neuroimmune processes as well.^18^

As a whole, the sensory nervous system is highly implicated in reflex arcs: neural pathways that control stereotyped motor responses to specific stimuli that do not require conscious thought. In contrast to unidirectional signaling in the ANS, signal transmission in the sensory nervous system is bidirectional. Classic reflex arcs consist of sensory (afferent) nerves that detect external stimuli and transmit sensory information to the spinal cord, from which it travels back down via motor (efferent) nerves and to organs such as the muscle to elicit behavioral outputs.^19^ Referred to as spinal reflexes, these include the withdrawal reflex and the knee-jerk reflex.^20^ Other reflexes, such as breathing and blinking, involve sensory inputs that go beyond the spinal cord and to the brainstem.^21,22^ All reflex arcs require both a sensory and a motor component; a motor output is typically generated or modified by sensory input. This demonstrates the highly coordinated nature of the sensory and motor branches of the PNS. Importantly, an emerging area of peripheral neuroimmunology is understanding not only physiologic but also neuroimmune reflexes along this highly integrated peripheral network.^23^

## 3. Adrenergic and cholinergic circuits in autonomic neuroimmunology

The ANS is a complex system and much of its anatomy goes beyond the scope of this review. For a more comprehensive description of the ANS, see McCorry 2007.^9^ Herein, we will highlight two autonomic circuits that represent the most well-known adrenergic and cholinergic systems in neuroimmunology: the splenic motor and vagal motor pathways.

### 3.1 Splenic motor pathway

The splenic motor nerve provides efferent sympathetic innervation to the spleen, a classic secondary lymphoid organ of the immune system.^24^ Sympathetic motor pathways consist of a preganglionic neuron, which originates in the brainstem or spinal cord, and a postganglionic neuron, which lies in autonomic ganglia and innervates target tissues. A single preganglionic neuron can synapse with postganglionic neurons in multiple effector tissues; this allows for coordinated sympathetic communication throughout the body. Interestingly, these sympathetic neurons utilize different neurotransmitters; preganglionic neurons use ACh whereas postganglionic neurons use NE. Upon release, NE binds to adrenergic receptors on effector tissues and initiates various signaling cascades. As such, sympathetic nerves like the splenic nerve are classified as being adrenergic in function.

The spleen receives sympathetic input from the celiac plexus, which houses neurons that release NE to splenocytes via the splenic nerve.^25,26^ As a large immune organ, the spleen harbors a broad range of immune cells.^27^ Indeed, it is well known that interruption of splenic nerve activity affects the levels of cytokines in circulation.^28,29^ However, the precise role of splenic motor neurons in regulating the immune system has yet to be closely examined.

### 3.2 Vagal motor pathway

The vagus nerve is a polymodal nerve that monitors the state of visceral tissues and regulates autonomic functions accordingly. Derived from the Latin word for “wandering,” the vagus is the longest nerve in the body.^30^ Its efferent motor neurons lie in the dorsal motor nucleus of the vagus and the nucleus ambiguus within the brainstem.^31^ These preganglionic fibers synapse on and release ACh to postganglionic cell bodies in the viscera of the thorax and abdomen. In contrast to the adrenergic nature of the sympathetic splenic nerve, the parasympathetic vagus nerve is cholinergic and regulates numerous internal physiologies including breathing, heart rate, and digestion.

### 3.3 The cholinergic anti-inflammatory pathway

**Fig. 4.**
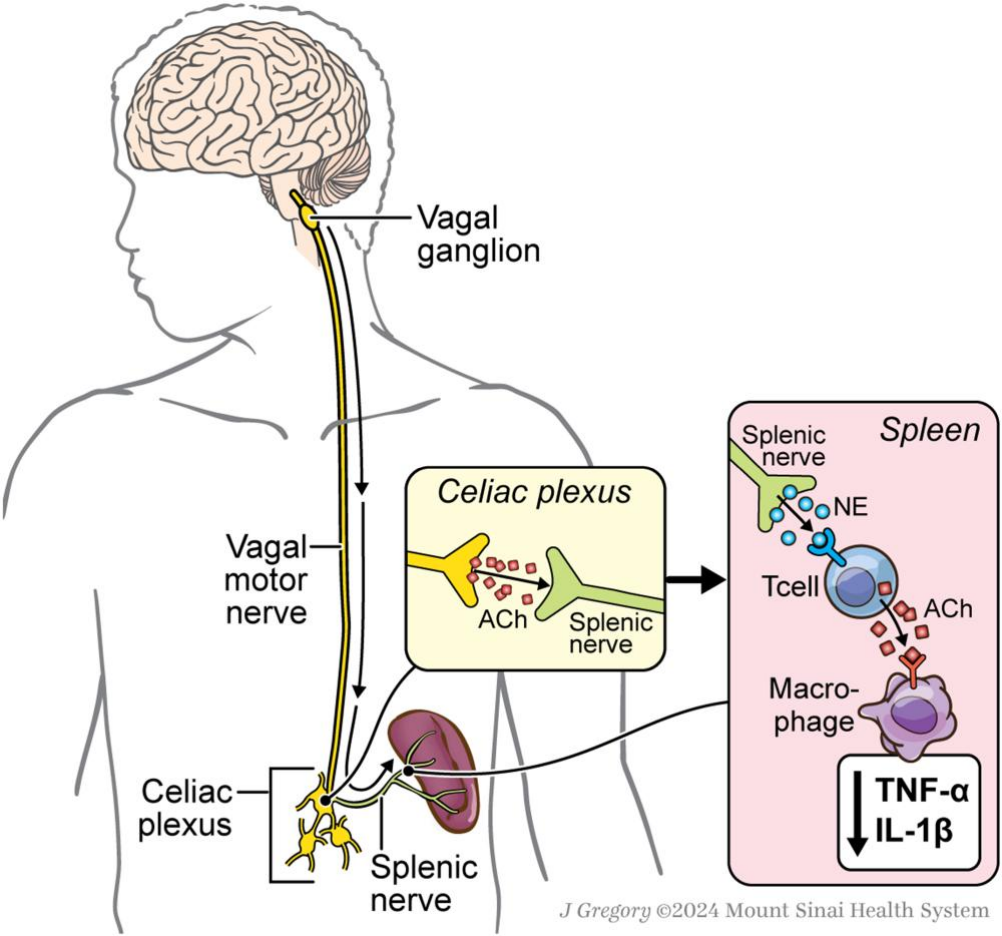
Circuitry of the cholinergic anti-inflammatory pathway (CAP). The CAP is a neuroimmune axis that serves to regulate systemic inflammation via the vagus nerve. Vagal motor neurons synapse in the celiac plexus, through which it intersects with the splenic nerve to modulate immune activity in the spleen. Upon activation of the vagus nerve and a resultant release of ACh, splenic nerves are stimulated to supply NE to T cells. In turn, these T cells synthesize and release ACh to macrophages, suppressing their production of the cytokines TNF-ɑ and IL-1β and limiting proinflammatory responses to maintain immune homeostasis.

A seminal study by Borovikova et al.^32^ showed that activation of the vagus nerve suppressed lipopolysaccharide (LPS)-induced systemic inflammation via the release of ACh; this was termed the cholinergic anti-inflammatory pathway (CAP). It was originally thought that ACh from vagal efferents directly activated macrophages to decrease the production of proinflammatory cytokines. However, further investigation revealed that these vagal neurons were not synapsing on macrophages. Rather, they converged upon neurons within the celiac plexus that then projected to the spleen via the splenic nerve; this suggested that vagus-derived ACh was modulating the splenic nerve instead of directly acting on macrophages (Fig. 4).^33^ Strikingly, despite its adrenergic nature, the splenic nerve activated a population of ACh-synthesizing T cells in the spleen that suppressed macrophage-derived cytokine production.^34^ In sum, CAP represents a complex neuroimmune circuit by which the ANS regulates systemic inflammation. How sensory cues influence this inflammatory reflex is now a major area of inquiry and will further inform how the sensory nervous system helps the body respond to changes brought about by disease.^35,36^ Moreover, many studies are now examining autonomic pathways that exist beyond the vagus and splenic nerves.^37–39^

## 4. Vagal neuroimmunology: peripheral sensation and neuroinflammation

Despite its involvement in one of the main autonomic circuits in neuroimmunology—the CAP—the vagus nerve has more sensory than motor functions. In fact, approximately 80% of vagal neurons are sensory and as many as 100,000 can be found in humans.^40,41^ The cell bodies of vagal sensory neurons lie in the jugular (superior) and nodose (inferior) ganglia of the vagus nerve.^42^ From there, they project to the brainstem as well as to visceral organs. These neurons have extensive functions throughout the body, all of which are critical to maintaining physiologic homeostasis.

### 4.1 Vagal sensory neurons as a gateway to sensory neuroimmunology

Sensory input, while not needed to activate the motor system, is necessary to adjust motor physiology when helping the body adapt to a changing environment. Sensory afferents thus represent the first arm of reflex arcs. Though reflexes serve as defense mechanisms, their motor outputs are also among the most common symptoms in sickness: coughing, vomiting, and diarrhea. Lung-innervating vagal sensory neurons were activated in LPS-induced pulmonary inflammation and in ovalbumin-induced allergic asthma in mice, demonstrating their role in detecting compromised airway integrity.^43,44^ In line with this finding, laryngeal water perfusion stimulated vagal neurons and evoked reflexes resembling cough in mice.^45^ Similarly, electrical stimulation of gastrointestinal vagal afferents in ferrets generated emetic responses, indicating that these afferents can relay sensory information from abdominal organs to generate reflexes.^46^ Vagotomy can suppress cough and emesis, further confirming the key role of vagal afferents in these responses.^47^ It has also been shown that vagal sensory neurons were activated by *Campylobacter jejuni*, a pathogen that causes diarrhea, and that diarrhea may be a side effect of vagal nerve stimulation.^48,49^ Taken together, these studies demonstrate that vagal sensory neurons critically regulate a host of motor physiologies.

Vagal sensory neurons are involved in neuroimmune circuits in which they can be stimulated by inflammatory cytokines. Early studies showed that vagal sensory neurons, which express receptors for tumor necrosis factor (TNF) and interleukin (IL)-1β, could be activated by microinjection or intravenous administration of these cytokines, respectively.^50,51^ Subsequently, it was found that disruption of vagal afferents by these cytokines could interfere with autonomic homeostasis.^52,53^ Most recently, IL-1β, IL-6, and IL-10 were shown to stimulate two distinct populations of vagal sensory neurons, one that regulated a proinflammatory response and another that regulated an anti-inflammatory response, respectively, following stimulation by LPS.^54^ Another recent study showed that LPS triggers sickness behavior by directly binding to Toll-like receptor 4 (TLR4) on vagal sensory neurons.^55^ Collectively, these studies have helped advance our understanding of how vagal sensory neurons respond to both cytokine and pathogen-associated cues to regulate systemic inflammation.

Vagal sensory neurons express large quantities of neuropeptides such as substance P (SP), calcitonin gene-related peptide (CGRP), and vasoactive intestinal peptide (VIP).^56^ While these neuropeptides act as bona fide neurotransmitters in the CNS, their efferent release into tissues have revealed their role as immune effectors.^57^ Therefore, although vagal sensory neurons are afferent in terms of sensory processing, they are efferent in terms of their immune functions. Indeed, it has been shown that vagal neurons within the nodose ganglion respond directly to IL-5 (afferent), which then triggers the release of VIP (efferent) to promote allergic inflammation.^58^ In the setting of infection, TRPV1^+^ vagal nociceptors, which are sensory neurons that detect noxious stimuli, were shown to suppress protective immunity. Specifically, susceptibility to *Staphylococcus aureus* pneumonia in mice is mediated by the inhibition of neutrophils and γδ T cells by sensory neuron-associated CGRP.^59^ Similarly, in the context of allergic lung inflammation, we have recently found that the CGRPβ isoform, which is less well understood than CGRPɑ, is activated by Janus kinase 1 (JAK1) within vagal sensory neurons to suppress inflammation.^60^ Although the vagus nerve is greatly appreciated for its ability to sense and regulate a variety of physiologic processes within the body, peripheral neuroimmunology is revealing how these neurons can also sense cytokine and microbial signals to directly and simultaneously coordinate tissue immunity and inflammation.

## 5. Somatosensory neuroimmunology and beyond

Similarly to vagal neurons, somatosensory neurons respond to a wide range of inflammatory cues, particularly within the skin. The skin is densely innervated by polymodal unmyelinated C-fibers that transmit a variety of sensory signals including heat, cold, pain, and itch.^61,62^ A major preconception in the field of somatosensation was that itch was simply a mild form of pain.^63^ However, it is now well understood that pain and itch are distinct sensations that utilize similar but different pathways.^64^ Further, itch biology has provided one of the clearest examples of how cytokine signals are directly linked to motor responses that promote a distinct mammalian behavior, namely scratching. Herein, we discuss how our understanding of pain has informed itch biology and detail how itch neuroimmunology has greatly enhanced our understanding of somatosensation while also directing future avenues of research into elucidating visceral neuroinflammation.

### 5.1 Pain

Pain is a protective mechanism defined as “an unpleasant sensory and emotional experience associated with, or resembling that associated with, actual or potential tissue damage.”^65^ This sensation typically results from the detection of noxious stimuli by nociceptors. When activated, nociceptors transmit pain signals through voltage-gated sodium, calcium, and potassium channels as well as TRPV1 and TRPA1.^66^ Though pain is primarily associated with the nervous system, the discovery that IL-1β could induce pain in vivo suggested that cytokines could directly activate and sensitize nociceptors.^52^ This finding was supported by the identification of other cytokines that could promote pain including TNF-α, IL-6, and IL-17A.^67^ Most cytokines that mediate pain are classically associated with autoinflammatory, autoimmune, or infectious processes (e.g. type 1, type 3, and/or Th17 immunity).^68^ These canonical immune processes are generally not associated with other sensations such as itch. Whereas cytokine blockade has not emerged as bona fide antinociceptive therapeutics, an understanding of how cytokines could activate sensory neurons was incredibly informative for itch biology.

### 5.2 Itch as a model of neuroimmune sensory arcs

Itch was first described as “an uncomfortable sensation that provokes a desire to scratch” by German physician Samuel Haffenreffer.^69^ Itch is mediated by specific neurons, referred to as pruriceptors, that respond to itch-inducing stimuli called pruritogens.^70^ Once a pruritogen binds to its receptor on a pruriceptor, there is a membrane depolarization mediated by nonselective cation channels including TRPA1 and TRPV1, which also regulate pain.^71^ Key discoveries in itch biology have shown that pruriceptors demonstrate itch specificity, in part by the neuropeptides that they produce such as brain natriuretic peptide and gastrin-releasing peptide, as well as the downstream signaling pathways they stimulate.^72,73^ However, the extent to which various factors can promote itch in response to signals in the periphery remains a major area of investigation.

Beyond neurotransmitters and neuropeptides, it is now well appreciated that cytokines are bona fide pruritogens and/or modulators of itch. The first cytokine associated with itch, IL-31, was identified within T helper type 2 (Th2) cells that promoted atopic dermatitis (AD)-like disease in mice.^74^ Indeed, it is well known that IL-31 induces scratching behavior when injected into mice and triggers calcium influx in sensory neurons.^75–77^ Subsequently, a number of type 2 inflammation-associated cytokines such as IL-4, IL-13, IL-33, and thymic stromal lymphopoietin were implicated in itch via their direct activity on sensory neurons.^78–81^ The cytokines IL-4, IL-13, IL-31, and downstream JAK1 are now major therapeutic targets for itch across a number of conditions including AD, prurigo nodularis, and chronic spontaneous urticaria.^82,83^

### 5.3 Mrgprs as itch receptors and mediators of neuroinflammation

Mas-related G protein-coupled receptors (Mrgprs), a family of GPCRs involved in somatosensation, have emerged as novel targets for itch.^84^ MrgprA3 and MrgprC11 in murine neurons were among the first identified itch receptors.^85^ However, other Mrgprs are expressed on immune cells; murine MrgprB2 and its human ortholog, MRGPRX2, demonstrate selective and/or high expression on mast cells.^86^ Given the role of mast cells in histaminergic itch, it was hypothesized that MrgprB2/MRGPRX2 could be implicated in itch. While mast cells are a rich source of histamine, it was found that activation of MrgprB2 triggers mast cells to promote nonhistaminergic itch as well.^87^ One of the most unique aspects of MrgprB2/MRGPRX2 biology is its emergence as a canonical receptor for SP. In addition to mediating itch, SP release from TRPV1^+^ nociceptors can be triggered by protease allergens, such as house dust mite, and activate MrgprB2 on mast cells.^88^ It has also been shown that SP can activate skin dendritic cells via MrgprA1 to promote Th2 cell responses.^89^ Collectively, the role of Mrgprs in itch remains a very exciting area of both basic and therapeutic exploration.

### 5.4 Skin inflammation

Beyond SP, it has been shown that CGRP release from sensory neurons can drive skin inflammation. In the setting of imiquimod-induced psoriasis-like disease in mice, it was shown that CGRP derived from sensory neurons expressing TRPV1 and Na_v_1.8 directly promoted psoriatic inflammation.^90–92^ In addition to sterile inflammation, bacterial proteases can directly activate sensory neurons to trigger the release of CGRP as well. This process results in the ability of skin pathogens to evade the immune response via CGRP-mediated suppression of neutrophil activity.^93^ Sensory neurons can also be specifically activated using chemo- or optogenetics. These technologies involve inserting synthetic receptors that respond to otherwise innocuous compounds or specific wavelengths of light, respectively, to broadly activate a cell of interest. Indeed, optogenetic activation of TRPV1^+^ sensory neurons demonstrated that CGRPα was a key driver of skin inflammation.^94^ More recently, skin sensory neuron-derived CGRP was found to regulate neutrophil and myeloid cell responses in the skin to promote optimal tissue regeneration.^95^ Collectively, these studies demonstrate again how neuropeptides function as neuronal effectors of immunity, inflammation, and tissue repair in both health and disease. It is very likely that the pleiotropic functions of CGRP are derived from very specific neuroimmune axes that arise in different inflammatory processes.

### 5.5 Gut inflammation

In contrast to the skin, which is innervated by somatosensory neurons, the gut is innervated by spinal visceral afferents which arise from the DRG as well. It is important to note that sensation from the intestinal tract is also supplied by the vagal sensory nervous system and the enteric nervous system (ENS), which likely has its own sensory components. For more information on the ENS, please refer to Rao and Gershon 2018 and Spencer and Hu 2020.^96,97^ Notwithstanding, many recent studies implicating neuroimmune mechanisms have centered on spinal sensory neurons from the DRG in relation to epithelial, stromal, and immune cells in the gut. These neurons, also classified as nociceptors, can generate visceral pain sensation via their expression of a wide range of ion channels that allow pain-related sensory information to be transmitted from throughout the gastrointestinal tract.^98,99^ Of particular interest in the context of gut inflammation are TRPV1, which has been linked to the mechanotransduction of visceral pain, and Piezo2, a bona fide mechanosensor that mediates sensation in response to mechanical distension.^100,101^ Accordingly, TRPV1-lineage nociceptors expressing the Piezo2 channel have been implicated in visceral hypersensitivity in the colon, supporting previous findings that link the expression of these channels to gastrointestinal dysfunction.^102–104^

Similarly to the skin, inflammation in the gut can be driven by local neuropeptide release from spinal sensory neurons. As previously noted, skin pathogens such as *Staphylococci* and *Streptococci* release bacterial toxins that can directly penetrate sensory neuron membranes to induce the release of neuropeptides.^93,105,106^ In contrast to pore-forming toxins in the skin, detection of *Salmonella enterica* serovar Typhimurium in the gut by nociceptors leads to the release of CGRP, which in turn modulates epithelial microfold (M) cells to promote mucosal protection.^107^ CGRP has also been found to activate immunoregulatory intestinal cells via a neuron-goblet axis to enhance gut protection in the setting of colitis.^108^ However, in contrast to the notion that toxins cause inflammation by damaging epithelial cells, *Clostridium difficile* toxin B (TcdB) directly binds to Frizzled receptors FZD1, FZD2, and FZD7 on gut-innervating sensory neurons and drives SP and CGRP release, resulting in colonic inflammation.^109,110^ This provides a new understanding of how neurons can drive inflammatory events in lieu of direct immune activation by pathogens or cytokines.

## 6. Concluding remarks

Classically, the CNS has been the major focus of neuroimmunology, particularly in autoimmune diseases such as multiple sclerosis and neurodegenerative conditions such as Alzheimer's disease. However, there is increasing appreciation for the PNS as a key player in orchestrating neuroimmune processes in peripheral tissues. Beyond even the capacity of the ANS to alter immune pathways via effectors such as neurotransmitters like ACh, there is tremendous interest in how the sensory nervous system both responds to and directs different immune functions across organs.

Sensory neurons arising from the DRG have been most well recognized for their somatosensory functions given the breadth of sensory modalities they relay including mechanoreception, thermoception, nociception, and pruriception. However, functions of the sensory nervous system transcend somatosensation and extend into immune regulation via vagal and spinal sensory neurons that widely innervate internal organs. Indeed, this system has evolved to detect immune signals from cytokines, bacteria, and other environmental irritants and coordinate responses accordingly. Therefore, the sensory system is the key regulator of reflex arcs that initiate protective responses to preserve tissue homeostasis. Furthermore, a tendency towards homeostasis exists across neuroimmune systems and is regulated by sensory innervation throughout the body.

The advent of new approaches and technologies has redefined our understanding of homeostatic physiology. Whereas sensory and motor functions were often viewed as separate modalities, peripheral neuroimmunology is increasingly demonstrating how reflex arcs that connect these two arms of the PNS synchronize neural and even immune balance throughout different organs. We speculate that there are many other reflexes triggered by the immune system to regulate immunity and inflammation and that they require uniquely orchestrated responses from the sensory and autonomic nervous systems. Peripheral neuroimmunology is an exciting field that has high potential to unveil transformative knowledge that can better inform the treatment of diseases of the nervous system, immune system, and beyond.

## Abbreviations

ACh: acetylcholine
AD: atopic dermatitis
ANS: autonomic nervous system
CAP: cholinergic anti-inflammatory pathway
CGRP: calcitonin gene-related peptide
CNS: central nervous system
DRG: dorsal root ganglion
ENS: enteric nervous system
GPCR: G protein-coupled receptor
JAK1: Janus kinase 1
LPS: lipopolysaccharide
Mrgpr: Mas-related G protein-coupled receptor
NE: norepinephrine
PNS: peripheral nervous system
SP: substance P
Th2: T helper type 2
TRP: transient receptor potential
VIP: vasoactive intestinal peptide
